# Editorial: Endocrine and Neuroendocrine Systems of Invertebrate Deuterostomes

**DOI:** 10.3389/fendo.2019.00755

**Published:** 2019-11-01

**Authors:** Honoo Satake, Dan Larhammar, Maurice R. Elphick

**Affiliations:** ^1^Bioorganic Research Institute, Suntory Foundation for Life Sciences, Kyoto, Japan; ^2^Department of Neuroscience, Uppsala University, Uppsala, Sweden; ^3^School of Biological and Chemical Sciences, Queen Mary University of London, London, United Kingdom

**Keywords:** neuropeptide, hormone, receptor, activity, invertebrate deuterostome

Invertebrate deuterostomes, which include echinoderms (e.g., sea urchins, starfish, and sea cucumbers) hemichordates (e.g., acorn worms), cephalochordates (e.g., amphioxus), and urochordates (e.g., ascidians), occupy an “intermediate” phylogenetic position with respect to vertebrates and protostomes (e.g., arthropods, nematodes, annelids, mollusks). Therefore, these animals are important model organisms in the fields of evolutionary biology, developmental biology, comparative physiology, and comparative genomics.

Until recently, relatively little was known about the endocrine and neuroendocrine systems of invertebrate deuterostomes by comparison with vertebrates and protostomes. However, over the past decade, advances in omics technology (genomics, transcriptomics, peptidomics, and metabolomics) have rapidly enhanced molecular characterization of low molecular weight neurotransmitters/hormones, neuropeptides, peptide hormones, and their receptors in invertebrate deuterostomes, which has facilitated investigation of their physiological roles. Endocrine and neuroendocrine systems play pivotal roles in the regulation of various biological processes, including reproduction, energy homeostasis, and feeding. To date, diverse neurotransmitters, steroid hormones, neuropeptides and peptide hormones, and their receptors have been characterized, and some of them have been shown to participate in regulation of a variety of biological processes in invertebrate deuterostomes. Furthermore, recent studies have also provided important insights into evolutionarily conserved and species-specific features of endocrine and neuroendocrine signaling systems and endocrine/neuroendocrine mechanisms, which reflect the evolutionary history and diversification of animal taxa.

This Research Topic includes five original research papers and two review articles that illustrate elegantly how our knowledge of the endocrine, neuroendocrine, and nervous systems of invertebrate deuterostomes has advanced recently. Taylor and Heyland reveal the induction of skeletogenesis by thyroid hormones (THs) in larvae of the sea urchin *Strongylocentrotus purpuratus*. The major TH, namely, 3,5,3',5'-Tetraiodo-L-thyronine (T4), triggers phosphorylation of extracellular signal-regulated kinase 1/2 in primary mesenchyme cells and epithelial cells in gastrulae, leading to the skeletal spicule formation. This is the first report of T4-mediated non-genomic pathways in a non-chordate invertebrate deuterostome. Wood et al. report localization of the expression of nine neuropeptide precursor transcripts in *S. purpuratus* larvae, providing a neuroanatomical basis for investigation of neuropeptide function in the larval nervous system of a model echinoderm and comparison with neuropeptide expression/function in other taxa. Perillo et al. report that orthologous genes of typical vertebrate pancreatic markers, such as *splox* and *spbrn1/2/4*, are expressed in neuronal precursor cells of *S. purpuratus* larvae. The authors also demonstrate that Splox regulates expression of a gene encoding the neuropeptide SpANP2 in Splox-positive neurons. These findings strongly support the view that vertebrate pancreatic endocrine cells and echinoderm larval neurons share gene regulatory mechanisms that already existed in neurons of the common ancestor of deuterostomes. Using the starfish *Asterias rubens* as an experimental model system, Tinoco et al. report the molecular characterization of a receptor for the neuropeptide NGFFYamide, which is orthologous to vertebrate neuropeptide-S (NPS) and the arthropod neuropeptide crustacean cardioactive peptide (CCAP). Furthermore, their analysis of NGFFYamide function in starfish revealed that it causes a delay in the onset of feeding, relaxes the body wall-associated apical muscle, induces tonic and phasic contraction of tube feet, and causes a decrease in locomotor activity. By comparing these effects of NGFFYamide in starfish with the effects of NPS in mammals and CCAP in arthropods, the authors speculate on the evolution of the physiological roles of a family of orthologous neuropeptides. Smith et al. report a metabolomic analysis of neurotransmitters, including gamma-aminobutyric acid (GABA), histamine, and serotonin, in the Crown-of-Thorns Seastar *Acanthaster planci*, revealing differences in neurotransmitter abundance associated with sex and feeding status. Furthermore, the biochemical analyses are complemented by immunohistochemical visualization of neurotransmitters in the radial nerve cords of *A. planci*.

In the first of two review articles, Mita provides an overview of the molecular characterization, functions, and species distribution and diversity of the starfish gonad-stimulating substance, relaxin-like gonad-stimulating peptide (RGP). Originally discovered 60 years ago but not chemically characterized until 10 years ago, RGP has a unique and special place in the history of echinoderm neuroendocrinology. In a second review article, Sekiguchi discusses the evolution of calcitonin/calcitonin gene-related peptide (CT/CGRP)-type signaling in deuterostomes and the characterization of CT/CGRP-type neuropeptides and their receptors in invertebrate chordates. It is inferred that the common ancestor of the chordates had a single gene encoding a CT/CGRP-type neuropeptide, with gene or genome duplication events then giving rise to multiple CT/CGRP-type neuropeptides in amphioxus and in vertebrates, respectively.

All of these articles shed light on the remarkable progress and broader scientific significance of studies of the endocrine, neuroendocrine, and nervous systems in invertebrate deuterostomes. However, in contrast to the growing body of studies on echinoderms and urochordates, experimental neuroendcrinology of hemichordates and cephalochordates remains largely uninvestigated. Furthermore, and perhaps not surprisingly, the focus of the articles in this Research Topic is on signaling molecules for which orthologs have been identified previously in vertebrates and/or protostomes. Largely unexplored are signaling systems that are uniquely associated with individual deuterostome phyla or a subset thereof. This reflects challenges associated with the elucidation of clade-specific ligand-receptor pairs. Recently, while the preparation of this Research Topic was in progress, Shiraishi et al. (1) reported machine learning-assisted systematic and efficient prediction and cell-based experimental validation of novel neuropeptide-receptor pairs in the urochordate *Ciona intestinalis*. The authors developed an original machine learning-based ligand-receptor predictor, and eventually identified 12 novel neuropeptide-receptor pairs. There now exists the exciting possibility of applying this research strategy to enable identification of novel neuropeptide-receptor partners in other invertebrate deuterostomes.

Finally, we express sincere thanks to all the authors, reviewers, and editors for their valuable contributions to this Research Topic.

## Author Contributions

HS, DL, and ME wrote the editorial.

### Conflict of Interest

The authors declare that the research was conducted in the absence of any commercial or financial relationships that could be construed as a potential conflict of interest.
